# Spatial Variation in Responses of Plant Spring Phenology to Climate Warming in Grasslands of Inner Mongolia: Drivers and Application

**DOI:** 10.3390/plants13040520

**Published:** 2024-02-14

**Authors:** Guang Lu, Mengchao Fang, Shuping Zhang

**Affiliations:** 1 Key Laboratory of Ecology and Environment in Minority Areas (National Ethnic Affairs Commission), Minzu University of China, Beijing 100081, China; 2 College of Life and Environment Sciences, Minzu University of China, Beijing 100081, China

**Keywords:** grassland, plant phenology, climate warming, precipitation, soil, spatial pattern, Inner Mongolia, husbandry community, prediction

## Abstract

Plant spring phenology in grasslands distributed in the Northern Hemisphere is highly responsive to climate warming. The growth of plants is intricately influenced by not only air temperature but also precipitation and soil factors, both of which exhibit spatial variation. Given the critical impact of the plant growth season on the livelihood of husbandry communities in grasslands, it becomes imperative to comprehend regional-scale spatial variation in the response of plant spring phenology to climate warming and the effects of precipitation and soil factors on such variation. This understanding is beneficial for region-specific phenology predictions in husbandry communities. In this study, we analyzed the spatial pattern of the correlation coefficient between the start date of the plant growth season (SOS) and the average winter–spring air temperature (WST) of Inner Mongolia grassland from 2003 to 2019. Subsequently, we analyzed the importance of 13 precipitation and soil factors for the correlation between SOS and average WST using a random forest model and analyzed the interactive effect of the important factors on the SOS using linear mixing models (LMMs). Based on these, we established SOS models using data from pastoral areas within different types of grassland. The percentage of areas with a negative correlation between SOS and average WST in meadow and typical grasslands was higher than that in desert grasslands. Results from the random forest model highlighted the significance of snow cover days (SCD), soil organic carbon (SOC), and soil nitrogen content (SNC) as influential factors affecting the correlation between SOS and average WST. Meadow grasslands exhibited significantly higher levels of SCD, SOC, and SNC compared to typical and desert grasslands. The LMMs indicated that the interaction of grassland type and the average WST and SCD can effectively explain the variation in SOS. The multiple linear models that incorporated both average WST and SCD proved to be better than models utilizing WST or SCD alone in predicting SOS. These findings indicate that the spatial patterns of precipitation and soil factors are closely associated with the spatial variation in the response of SOS to climate warming in Inner Mongolia grassland. Moreover, the average WST and SCD, when considered jointly, can be used to predict plant spring phenology in husbandry communities.

## 1. Introduction

Climate warming has become the primary changing environmental factor affecting natural ecosystems [1,2]. Phenology is a rhythmic phenomenon influenced by natural biological and abiotic processes in response to periodic changes in climate conditions. Examples include plant green-up and the melting of ice and snow [3,4]. Global climate change has induced extensive phenology changes in nature [5,6]. These changes not only disrupt interspecific relationships, such as nutrition and symbiosis formed during species evolution [7], but also impact biodiversity and the dynamic balance of ecosystems by influencing material cycles and energy flow [8,9,10]. Plant phenological change is an important component of research on the ecological effects of climate warming. The growth period of plants directly affects the balance of terrestrial ecosystems [11,12], and the start date of the plant growth season (SOS) is closely related to changes in air temperature, representing an independent phenological feature in the ecosystems responding to climate change [13,14,15,16,17]. 

Grasslands distributed in the Northern Hemisphere are a type of vulnerable vegetation where the SOS is particularly sensitive to climate warming [18,19]. Additionally, grasslands play a crucial role in animal husbandry, making the impact of climate warming on the plant growth season in these areas closely linked to the livelihoods of local communities. Plant growth in grassland ecosystems is influenced not only by air temperature but also by precipitation and soil factors [20,21,22]. Snow cover significantly affects SOS in grasslands by contributing to soil water supply through snow melting and increasing soil temperature in winter [23,24,25,26]. The reduction in snow cover days (SCD) can result in soil freezing and plant root death, consequently delaying SOS [27,28]. In addition, soil nutrients also influence plant phenology. For instance, soil nitrogen affects the plant phenology of grassland ecosystems on the Tibetan Plateau [22]. Studies on forest ecosystems indicate that the influence of climate on plant growth is more pronounced in the soil organic layer. Changes in soil nutrients due to long-term precipitation alterations in the soil organic layer affect the seasonal trajectories of fine root biomass [29]. Therefore, it is necessary to understand the integrated effects of precipitation and soil factors on the response of SOS to climate warming in grasslands. 

The precipitation and soil factors often display spatial and temporal variations [30,31,32]. For example, SCD increased significantly in the central Andes of Chile and Argentina between 2000 and 2016 [33], while it decreased in the Mongolia Plateau between 1982 and 2015 [34]. Continental-scale studies conducted in Asia, North America, Europe, and the Northern Hemisphere revealed that SCD increases with increasing altitude [31], and the response of SCD to climate warming varies along latitudinal gradients [35]. Regional-scale studies have demonstrated that SCD increases with the increasing altitude in the Alps [36], and it increases with latitude in northeast China [37]. The soil factors also exhibit significant spatial heterogeneity. Altitude, gradient, and the roughness of terrain show significant correlations with soil organic matter and total nitrogen [38]. Therefore, the spatial heterogeneities of precipitation and soil factors may induce spatial variation in the sensitivity of SOS responding to climate warming. 

Despite numerous studies demonstrating spatial variation in the response of SOS to climate warming [4,14,18], most of these studies were conducted at the continental scale and did not consider the integrated effects of precipitation and soil factors [39]. Grassland ecosystems are regionally distributed, such as the Eurasian grassland located in the north of the Eurasian continent [34,40]. Regional-scale and multi-factor research is more precise for understanding the sensitivity of SOS responses to climate warming in grassland ecosystems. Therefore, it is necessary to investigate the spatial variation in grassland SOS response to climate warming and the effects of precipitation and soil factors on such variation at regional scales. 

Inner Mongolia, China, located on the Eurasian grassland [40,41], features diverse grassland types, including meadow grasslands, typical grasslands, and desert grasslands, distributed from east to west. The annual precipitation for these grassland types varies, with meadow grasslands receiving 400–450 mm, typical grasslands receiving 300–350 mm, and desert grasslands receiving 150–200 mm. Snow cover is present throughout the region in winter. Positioned between 37°34′ N and 53°23′ N, Inner Mongolia exhibits altitudes ranging from 90 to 3300 m [42]. This spatial variation in precipitation and soil factors makes Inner Mongolia a suitable case for investigating the spatial variation in the Start-of-Season (SOS) response to climate warming and its drivers in grasslands. Moreover, the grasslands of Inner Mongolia serve as crucial pastures, linking the response of SOS to climate warming directly to the livelihoods of local communities. Establishing SOS prediction models for local pastoral communities is imperative, requiring a regional scale and multi-factor approach. In this paper, we conducted an analysis of the spatial variation in the responses of SOS to climate warming in Inner Mongolia from 2003 to 2019, utilizing MOD13A1 and MYD13A1 data. We also examined the effects of precipitation and soil factors on such variation. Based on the results of driver analyses, we established models to predict SOS in pastoral areas within meadow, typical, and desert grasslands, respectively. These models aim to optimize grazing management in husbandry communities.

## 2. Results

### 2.1. Spatial Pattern of Temporal Trends in SOS and Average Winter–Spring Air Temperature 

**Figure 1:**
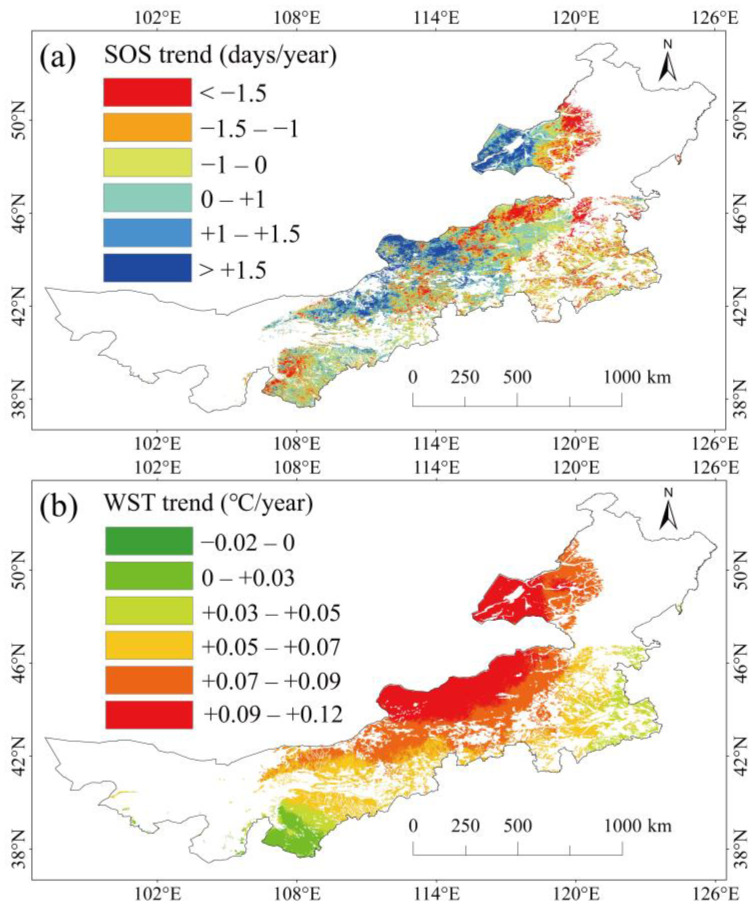
Spatial pattern of temporal changes from the start date of the plant growth season (SOS) (**a**) and average winter–spring air temperature (WST) (**b**) in Inner Mongolia grassland from 2003 to 2019. “+” in (**a**) indicates delay, “–” indicates advance; “+” in (**b**) indicates rise, “–” indicates decrease.

Trends in SOS (Figure 1a) and average winter–spring air temperature (WST) (Figure 1b) in Inner Mongolia grassland from 2003 to 2019 exhibited evident spatial heterogeneity. The SOS was significantly advanced in 32% of the pixels and significantly delayed in 21% of the pixels. The change rates of SOS in meadow grasslands, typical grasslands, and desert grasslands were −1.06 days/year, −0.27 days/year, and +0.56 days/year, respectively. The average WST displayed an increasing trend in 99.9% of the study area, with significant spatial variations (Figure 1b). The increasing rates of average WST for meadow grasslands, typical grasslands, and desert grasslands were 0.073 °C/year, 0.072 °C/year, and 0.078 °C/year, respectively.

### 2.2. Spatial Pattern of the Correlation between SOS and Average WST

**Figure 2:**
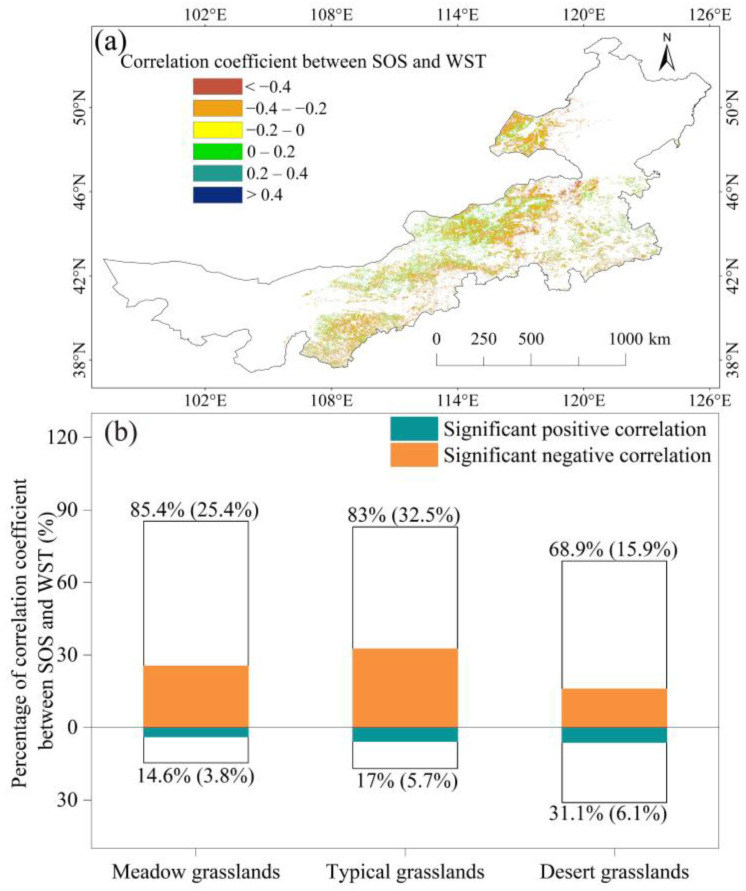
Spatial patterns of the correlation coefficient between SOS and average winter–spring air temperatures (WST) for Inner Mongolia grassland from 2003 to 2019 (**a**), and the pixel percentage of negative and positive between SOS and average winter–spring air temperature (WST) correlation coefficients in the meadow, typical and desert grasslands (**b**). The percentage of pixels with significant correlations is denoted between parentheses.

The proportion of pixels exhibiting a significant correlation between the SOS and average WST accounted for 34.2% of the total pixels (*p* < 0.05). Within this, 28.7% displayed a negative correlation, while 5.5% exhibited a positive correlation (Figure 2a). The areas with negative correlation coefficients in meadow and typical grasslands were higher compared to desert grasslands, with percentages of 85.4%, 83%, and 68.9% for the three types of grassland, respectively (Figure 2b). The areas with significant negative correlation coefficients in meadow and typical grasslands were also higher compared to desert grasslands, with percentages of 25.4%, 32.5%, and 15.9% for the three types of grasslands, respectively (Figure 2b). Conversely, the positive correlation percentage was higher in desert grasslands compared to meadows and typical grasslands.

**Figure 3:**
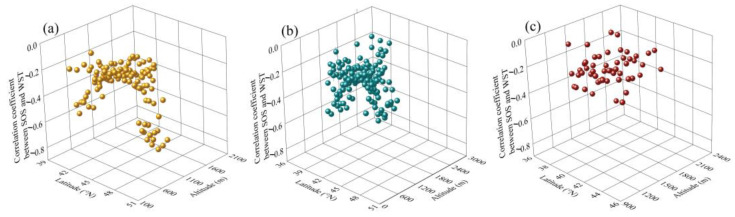
Altitude and latitude distribution of the pixels with a significant negative correlation between SOS and WST in meadow grasslands (**a**), typical grasslands (**b**), and desert grasslands (**c**). Dots represent values with significant negative correlation between SOS and WST.

The pixels with a significant negative correlation coefficient between SOS and WST were distributed in the 39° N~51° N and 300 m~2100 m ASL meadow grasslands (Figure 3a), in the 37° N~50° N and 400 m~2800 m ASL typical grasslands (Figure 3b), and in the 37° N~46° N and 900 m~2300 m ASL desert grasslands (Figure 3c).

### 2.3. Effects of Precipitation and Soil Factors on the SOS–WST Correlation

**Figure 4:**
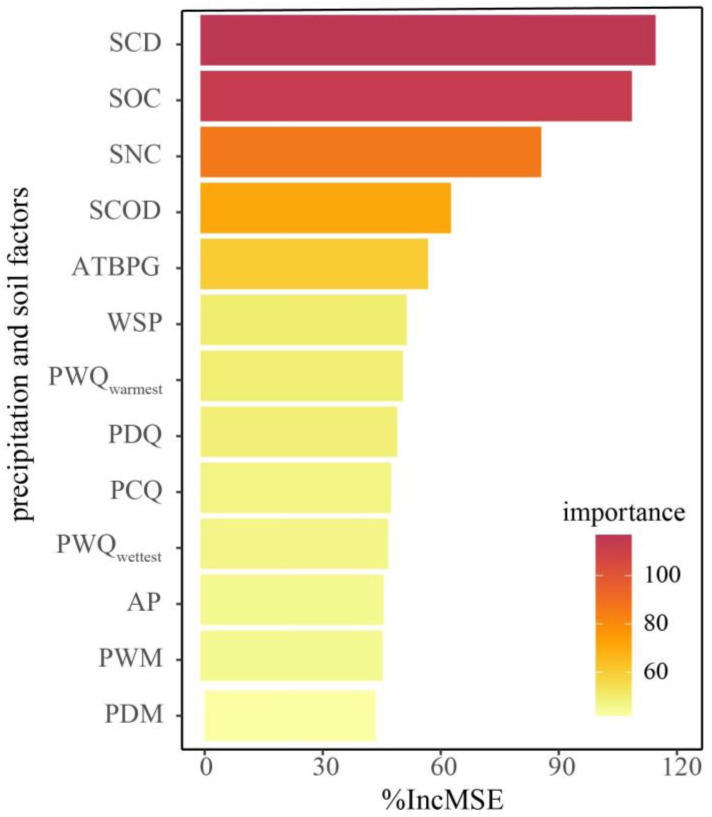
The effects of precipitation and soil factors on the correlation coefficient between SOS and average WST in a random forest model. SCD: snow cover days, SNC: soil nitrogen content, SOC: soil organic carbon content, SCOD: snow cover onset date, ATBPG: the average temperature of the seven days before the onset of plant growth, PDM: precipitation of driest month, PWQ_warmest_: precipitation of warmest quarter, WSP: winter and spring precipitation, PCQ: precipitation of coldest quarter, PWM: precipitation of wettest month, AP: annual precipitation, PDQ: precipitation of driest quarter, and PWQ_wettest_: precipitation of wettest quarter.

The results of the random forest model indicate that the average %IncMSE of the 13 factors was 63.29%. Among these, the factors with %IncMSE values higher than the average of the 13 factors included snow cover days (SCD), soil nitrogen content (SNC), and soil organic carbon content (SOC). Notably, SCD had the highest %IncMSE at 115.67% (Figure 4).

### 2.4. Spatial Patterns of SCD, SNC and SOC in Inner Mongolia Grassland

**Figure 5:**
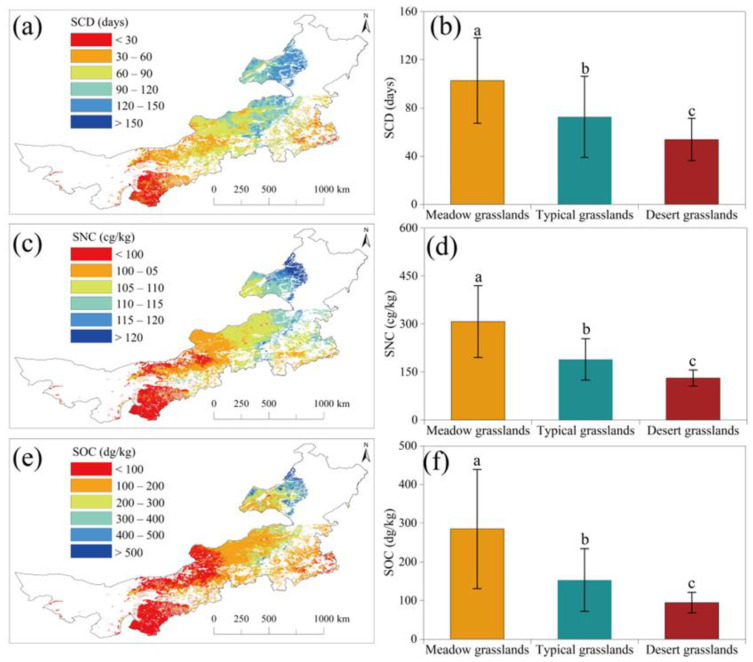
Spatial pattern of SCD, SNC, and SOC in Inner Mongolia grassland (**a**,**c**,**e**) and the differences of SCD, SNC, and SOC for different types of grasslands (**b**,**d**,**f**). SCD: snow cover days, SNC: soil nitrogen content, SOC: soil organic carbon content. The values of a, b, and c up the bars indicate significant differences between the groups.

The average SCD from 2003 to 2019 exhibited significant variations across different types of grassland (Figure 5a). Meadow grasslands had the longest average SCD (103 days/year), while desert grasslands had the shortest (54 days/year) (Figure 5b). Similarly, the average SNC and SOC from 2016 to 2019 displayed significant variations across different types of grasslands (Figure 5c,e). Meadow grasslands showed the highest SNC and SOC values (307.38 cg/kg and 284.89 dg/kg, respectively), while desert grasslands showed the lowest values (131.09 cg/kg for SNC and 94.5 dg/kg for SOC) (Figure 5d,f).

### 2.5. The Interactive Effects of Grassland Type, WST, SCD, SNC, and SOC on SOS

Based on the results of the random forest model and the spatial patterns of important factors, linear mixed models (LMMs) were established using all the single variables and interactions of grassland type, WST, SCD, SNC, and SOC on SOS as explanatory variables. Ranked in ascending order of AIC values, the top seven models are listed in Table 1. The results show that the model with the interaction of grassland type, WST, and SCD had the smallest AIC value (Table 1), suggesting that the interaction of grassland type, WST, and SCD effectively explains the variation in SOS.

### 2.6. Models for Predicting SOS in Sampled Pastoral Areas

Based on the LMM with the smallest AIC value, WST and SCD were selected as factors to establish models for predicting SOS in sampled pastoral areas using multiple linear regression (Table 2) and simple linear regression. The optimal models, selected based on the criteria of R^2^ > 0.5, VIF < 10, and the smallest RMSE and MAE, are as follows: meadow grasslands, SOS = −6.778 WST − 0.305 SCD + 79.873; typical grasslands, SOS = −21.046 WST − 0.279 SCD + 5.146; and desert grasslands, SOS = −13.647 WST − 0.178 SCD + 47.640 (Table 2).

## 3. Discussion

Global warming has led to the continuous advancement of SOS in various regions of the Northern Hemisphere [43,44]. Our findings indicate a warming trend in Inner Mongolia grassland during the period of 2003–2019, particularly in the northeast. The region experiencing SOS advancement surpasses the area experiencing an SOS delay in Inner Mongolia grassland. Previous research on the Mongolian Plateau showed that SOS advanced by 0.3 days/year from 2001 to 2017 [45]. In contrast, our study found that SOS in Inner Mongolia grassland advanced by 0.18 days/year from 2003 to 2019. Similarly, our results differ from those of Sa et al. concerning the Mongolian Plateau (0.39 days/year) [46]. These disparities may result from the following main reasons. Firstly, variations in the models used to estimate SOS in different studies are given as follows: we employed the S-G model in TIMESAT to simulate the NDVI curve, while Sa et al. used a logistic function. The S-G model automates the NDVI curve’s simulation and SOS extraction, reducing errors associated with the manual simulation of the NDVI curve [47]. Secondly, differences in the research areas might contribute to existing studies’ cover of the Mongolian Plateau (including Inner Mongolia of China and Mongolia), while our research specifically focuses on Inner Mongolia. The latitude of Mongolia is higher, and the area of steppe grasslands is larger than in Inner Mongolia [48]. Thirdly, considering the fact that available studies and our study were conducted using a relatively short time series, the use of different time windows for analyses may also result in variations in SOS. These differences may contribute to distinctions between our results and those reported in existing studies. Additionally, the temporal change trend of SOS in Inner Mongolia grassland in this study was less than the 0.38 days/year found in the Loess Plateau [49] and the 0.19 days/year found in Xinjiang [50]. Therefore, it underscores the importance of conducting plant phenology responses to climate change on a regional scale.

It is noteworthy that the SOS in desert grasslands exhibited a delayed trend from 2003 to 2019. In these areas, the average increase rate of the average WST was 0.078 °C/year. The proportion of pixels showing a significant positive correlation between SOS and average WST accounted for 6.1% of the pixels with significant correlations. This percentage was higher than that observed in meadows and typical grasslands, suggesting that the SOS in certain areas of desert grasslands may be delayed due to rising air temperatures. The SOS in desert grasslands is influenced not only by air temperature but also by soil water content [51]. The highest rate of increase in air temperatures in desert grasslands, coupled with increased evaporation, leads to a reduction in soil water content and consequent delays in SOS. Simultaneously, the limited SCD in desert grasslands contributes to soil freezing and plant root death, further contributing to the delayed SOS [27,28].

Despite the evident warming and SOS advancement trends in Inner Mongolia grassland from 2003 to 2019, only 28.7% of the total pixels exhibited a significant negative correlation between SOS and the average WST. This highlights the spatial variation in the response of SOS to climate warming. Therefore, it is necessary to analyze the interaction of average WST and other environmental factors on the SOS. The higher percentage of negative correlation in meadow and typical grasslands suggests that SOS is more sensitive to climate warming in these areas compared to desert grasslands. This spatial variation underscores the importance of considering variations in precipitation and soil properties. Results from the random forest model indicate that precipitation and soil factors influence the response of SOS to climate warming, with SCD identified as the most critical factor. SCD reflects winter precipitation, contributing to increased soil water and the maintenance of soil temperature [25,26]. Warm and moist soil conditions are beneficial for plant growth by promoting plants to green up and increasing the net photosynthetic rate [52,53]. The spatial pattern of SCD reveals that SCD is highest in meadow grasslands and lowest in desert grasslands. As precipitation gradually decreases from east to west in Inner Mongolia [54], the meadow and typical grasslands in the northeast experience more snow cover in winter, maintaining higher soil temperature and humidity. The combined effect of warm air and moist soil promotes plant growth in meadows and typical grasslands, making the SOS response to climate warming more sensitive in these regions. Conversely, the central and western regions of Inner Mongolia, characterized by an arid or semi-arid climate with less snow cover, experience a shorter SCD [40]. This condition is less conducive to plant growth in desert grasslands in the central and western regions, resulting in the less sensitive response of SOS to climate warming in this grassland type. Our results regarding the altitude distribution of pixels with a significant negative correlation between SOS and WST show that most of the pixels of desert grasslands are distributed in relatively higher altitude (above 2000 m ASL) areas, which may be associated with the increase in SCD with altitude [31]. 

While numerous studies have explored the impact of terrain on the relationship between SOS and climate change [6,13,23], there has been limited focus on the influence of soil factors on this relationship [39]. Since soil nutrients play a crucial role in plant growth [20] and the spatial variation in soil factors is significant [38], it becomes essential to consider the effects of soil factors on the correlation between SOS and air temperature. The results from our random forest model highlight the significance of SOC and SNC in influencing the response of SOS to average WST. SNC promotes photosynthesis and accelerates plant growth [55], while SOC supports plant root growth and facilitates nutrient uptake [56]. Our results show that SNC and SOC in Inner Mongolia grassland exhibit clear spatial differences (Figure 5), decreasing from northeast to southwest. Meadow grasslands have significantly higher SNC and SOC compared to typical and desert grasslands. Additionally, typical grasslands exhibit higher SNC and SOC levels than desert grasslands. Soil that is both moist and nutrient-rich is favorable for plant growth [57]. Moreover, the abundance of SOC and SNC influences the species composition of the plant community, affecting plant phenology due to the differences among species [58]. In meadow grasslands, dominant plant species include *S. baicalensis*, *L. Chinensis*, and *Cleistogenes mucronata*. In desert grasslands, dominant species comprise *S. Krylovii*, *S. bungeana,* and *A. ordosica*. The growth of species in meadow grasslands requires better hydrothermal conditions than those in desert grasslands [21]. Consequently, the differences in soil nutrients and plant species composition contribute to a more sensitive response of SOS to climate warming in meadows and typical grasslands compared to desert grasslands. Despite the importance of SNC and SOC, the results of LMMs show that the interaction of grassland types, WST, and SCD exhibits the smallest AIC value. Why do the models, including SOC, and SNC have a higher AIC? The fact that the spatial pattern of grassland types aligns with the patterns of SNC and SOC may be an important reason. The spatial pattern of grassland types represents the effects of SNC and SOC in the model with the smallest AIC. 

The SOS is intricately linked to the grazing plans of husbandry communities. The ability to predict the plant growth date is crucial for effective grazing management. In Inner Mongolia, local communities typically commence grazing approximately 15 days after green up, ensuring optimal grass growth and preventing the degradation of grasslands [59]. Therefore, determining the onset of plant growth in spring is critical for establishing the appropriate grazing time. Local residents traditionally rely on their observations on the grass green-up date for the previous two or three years to gauge the start date of plant growth in the current year [59]. However, climate change may disrupt these traditional observations. To mitigate the impact of climate change, communities have had to purchase more forage grass to sustain their animals, leading to increased economic costs [60]. Based on the LMM with the smallest AIC, models that incorporate WST and SCD can be established in three types of grasslands, respectively. Our results in Table 2 show that the models incorporating both WST and SCD have the smallest RMSE and MAE values in three types of grasslands, which indicates that the prediction models for SOS using both WST and SCD are better than those using WST or SCD alone. Additionally, the coefficients of these models on three types of grasslands are different, which suggests that the prediction model should be established based on the grassland type in which the pastoral community is located. The local animal husbandry departments can use long-term local SOS, WST, and SCD data can establish models for predicting SOS and continuously record annual SOS, WST, and SCD data to refine the models. In future research, the effectiveness of these models should also be tested by comparing them with the traditional methods of local husbandry people. 

## 4. Materials and Methods

### 4.1. Study Area

**Figure 6:**
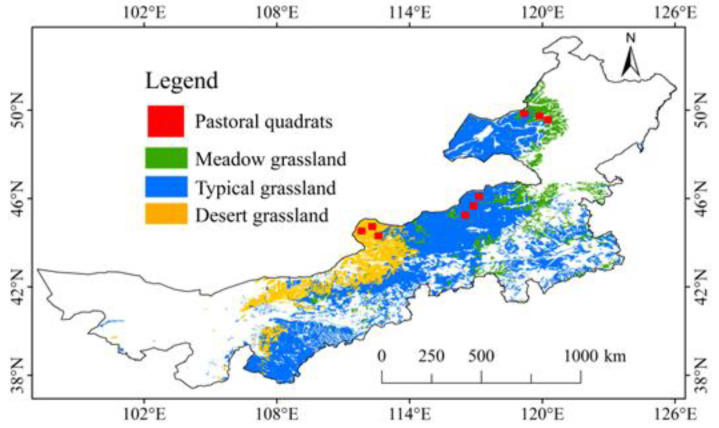
Spatial pattern of grassland types and distribution of pastoral quadrats in Inner Mongolia.

Our study site was Inner Mongolia grassland, China’s mid-northern region, between 37°34′ N~53°23′ N and 97°12′ E~126°04′ E, covering an area of 866,700 km^2^ (Figure 6). The altitude ranges from 90 to 3300 m. Inner Mongolia grassland forms a transitional zone between arid and semiarid areas in the northwest of China. Conditions in winter are cold and dry, and in summer, it is warm and wet [61,62]. The annual average air temperature is 5.5 °C [63]. Annual precipitation gradually decreases from approximately 400 mm in the east to about 200 mm in the west, resulting in a transition in grasslands from meadow, typical, and desert grasslands from east to west [42] (Figure 6). The dominant plant species in meadow grasslands are *S. baicalensis*, *L. Chinensis*, and *Cleistogenes mucronata* [64,65]. The dominant species in typical grasslands are *Stipa grandis* and *Leymus chinensis* [65]. The dominant plant species in desert grasslands are *S. Krylovii*, *S. bungeana*, and *A. ordosica* [64].

### 4.2. Research Design

Firstly, we analyzed the spatial pattern of the SOS variation trend and the average WST variation trend from 2003 to 2019 for Inner Mongolia grassland. Secondly, we examined the spatial pattern of correlation coefficients between the SOS and average WST from 2003 to 2019 in Inner Mongolia grassland, representing the spatial distribution of the SOS response to climate warming. Thirdly, we investigated the effects of nine precipitation factors and two soil factors on the correlation coefficients between the SOS and average WST. This was accomplished using a random forest model to identify the most influential environmental factors. Subsequently, we established linear mixed models (LMMs) to analyze the interactive effects of grassland types, WST, and the important environmental factors identified by the random forest model on SOS. Finally, we selected three 10 km × 10 km pastoral community quadrats in each type of grassland to establish models for predicting the SOS in the pastoral areas (Figure 6). Multiple linear regression models and simple linear models, utilizing the factors identified in the optimal LMM, were then established for the SOS prediction in pastoral areas of different grassland types.

### 4.3. Data Acquisition

#### 4.3.1. Grassland Types

The permanent grassland area in Inner Mongolia from 2003 to 2019 was derived from the MCD12Q1 product, which was generated by the International Geosphere Biosphere Program (IGBP) [66], with a spatial resolution of 500 m and was downloaded from the Land Process Distributed Activity Archive Center of the United States Geological Survey (USGS) “https://appeears.earthdatacloud.nasa.gov/ (accessed on 27 May 2020)”. We intersected the permanent grassland area in Inner Mongolia from 2003 to 2019 with the 1:500,000 grassland resource distribution map in China “https://www.resdc.cn/data.aspx?DATAID=355 (accessed on 5 July 2020)” and determined the three main types of grasslands in Inner Mongolia.

#### 4.3.2. SOS Data

Normalized Difference Vegetation Index (NDVI) data are suitable for use to calculate the SOS [67]. We used the dynamic threshold method to extract SOS, which defines the SOS as the corresponding date when NDVI rises to a certain threshold [68]. In this study, the SOS is defined as the date when the NDVI value reaches 20% of its amplitude. We selected the NDVI datasets of MOD13A1 and MYD13A1 (version 6) from MODIS to extract the SOS of Inner Mongolia grassland from 2003 to 2019. The dataset had a spatial resolution of 500 m and a time interval of 16 days (https://ladsweb.nascom.nasa.gov; (accessed on 27 May 2020)). The quality layer of MOD (MYD) 13A1 NDVI data was used to remove low-quality pixels. We loaded NDVI into the TIMESAT software for SOS extraction [69]. We removed the negative value of NDVI because it indicates that there are no green plants. The dual logistic function and median filter (Spike value = 0.5) were used to remove abnormal values. To estimate the SOS, an amplitude threshold of 0.2 was selected to account for varying ground phenology characteristics [70].

#### 4.3.3. Average WST Data

The average WST was calculated as the monthly average air temperature data from November of the previous year to April of the current year. Monthly average air temperature data were sourced from “the monthly average air temperature dataset with a 1 km resolution in China from 1901 to 2020” at Loess Plateau Sub Center, National Earth System Science Data Center, National Science & Technology Infra-structure of China (http://loess.geodata.cn; accessed on 27 May 2020).

#### 4.3.4. Precipitation and Soil Data

SCD is calculated using MODIS MOD10A1 and MYD10A1 (http://nsidc.org; accessed on 20 June 2020)). Cloud pixels need to be eliminated from the original MODIS snow data to accurately calculate SCD. To perform this, we first combined MYD10A1 and MOD10A1 data using the maximum synthesis method to reduce some of the cloud pixels [71]. The data without cloud from the previous day (or the next day) were then used to substitute the data from the day with cloud coverage, and the four–neighbor–pixel method was used to further remove the cloud pixels [72]. A cloud layer pixel is classified as a snow layer pixel if at least three snow layer pixels are present in the four adjacent pixels [73]. The snow cover phenology parameters were calculated as follows:

$$SCD=\sum_{i=1}^{n}\left(S_{i}\right)$$

where *n* represents the total number of days in a year; *S_i_* denotes a non-snow or snow pixel, with a value of 0 or 1, respectively.

The precipitation data were sourced from the Global Climate and Weather Dataset (https://www.worldclim.org/; accessed on 20 June 2020), including the precipitation of the coldest quarter (PCQ), precipitation of the warmest quarter (PWQ_warmest_), precipitation of the driest quarter (PDQ), precipitation of the wettest quarter (PWQ_wettest_), precipitation of the driest month (PDM), precipitation of the wettest month (PWM), annual precipitation (AP), winter and spring precipitation (WSP), the snow cover onset date (SCOD), and the average temperature of the seven days before the onset of plant growth (ATBPG). Soil data were sourced from the Soil Grid (https://soilgrids.org/; accessed on 20 June 2020), including soil organic carbon (SOC) and soil nitrogen content (SNC). The area data were converted to point data for subsequent analysis. We loaded all the area data into ArcGIS and used the “raster to point” tool to convert area data to point data.

### 4.4. Statistical Analysis

The least square method was used to extract the temporal trend of SOS and average winter–spring air temperature at the pixel level from 2003 to 2019 [74]. The formula is as follows:

$$Slope=\frac{n\sum_{i=1}^{n}iX_{i}-\sum_{i=1}^{n}i\sum_{i=1}^{n}X_{i}}{n\sum_{i=1}^{n}i^{2}-{\left(\sum_{i=1}^{n}i\right)}^{2}}$$

where *i* is from 1 to *n*, *n* is the total number of years, and *X_i_* is the SOS or average winter–spring air temperature of the *i*th year. A *slope* < 0 indicates a downward trend, while a *slope* > 0 indicates an upward trend. 

Pearson correlation analysis was used to assess the relationship between the SOS and average WST at the pixel level. The *t*-test was used to examine the significance of change trends in the SOS and average WST, as well as the significance of the correlation between the SOS and average WST. The change trend and correlation were considered significant when *p* < 0.05.

A random forest model was utilized to identify the precipitation and soil factors significantly affecting the correlation coefficients between the SOS and average WST. Random forest is a multivariate analysis method that ranks the relative importance of environmental factors, effectively avoiding overfitting and collinearity. The relative importance of an environmental factor is determined by the increase in prediction error when the data for that factor are replaced [75]. The Percentage of Increased Mean Square Error (%IncMSE) measures the decrease in prediction accuracy when the values of an environmental factor are replaced with random numbers [76]. An environmental factor with a %IncMSE value higher than the average %IncMSE value of all factors is considered important [77]. In this study, the dependent variable is the correlation coefficient between the SOS and average WST, and the independent variables include the following 13 precipitation and soil factors: PCQ, PWQ_warmest_, PDQ, PWQ_wettest_, PDM, PWM, AP, WSP, SCOD, ATBPG, SCD, SNC, and SOC. The model was established using the “random forest package” in R software [78]. Based on the result of the random forest model, linear mixed models (LMMs) were established to determine the interactive effect of important factors on SOS. Different interactive factors between grassland types and important environmental factors were used as fixed factors, with pixels as random factors. The model with the smallest AIC value was selected as the better model compared to others. LMMs were established in SPSS (20.0), and the significance level was set at 0.05.

The important factors identified in the optimal LMM were used to establish the SOS prediction models. The pixels in the quadrats were converted into points, and MODIS data of the SOS, average WST, and the factors in the optimal LMM at each point were extracted. Eighty percent of the points were used to establish the prediction model, and 20% of the points were used to test the predictability of the models. Multiple linear regression was used to establish prediction models with two independent variables. Simple linear regression was used to establish models with one independent variable. The multi-collinearity of two or more environmental factors in the model was tested using the variance inflation factor (VIF). VIF > 10 indicates high collinearity [79]. The predictability of the models was evaluated using the root mean square error (*RMSE*) and mean absolute error (*MAE*) [80].

$$RMSE=\sqrt{\frac{\sum_{i=1}^{n}{\left(O_{i}-P_{i}\right)}^{2}}{n}}$$

$$MAE=\frac{1}{n}\sum_{i=1}^{n}\left|O_{i}-P_{i}\right|$$

where *n* is the number of data, *O_i_* is the *i*th observed values, and *P_i_* is the *i*th predicted values. The model with R^2^ > 0.5, VIF < 10, and the smallest *RMSE* and *MAE* values were selected as the prediction model for SOS using the “stats” package in R. 

## 5. Conclusions

Temporal variations in plant spring phenology and average winter–spring air temperature from 2003 to 2019 exhibit spatial heterogeneity in Inner Mongolia grassland. There are spatial differences in the response of the SOS to climate warming. Snow cover days, soil organic carbon, and nitrogen content, which show a significant spatial pattern across different types of grassland, are the main factors related to such spatial variation. The interaction terms of the average winter–spring air temperature and SCD have the greatest influence on the SOS for different types of grasslands. The binary linear model composed of average winter–spring air temperature and SCD can predict the SOS effectively. MODIS NDVI, average winter–spring air temperature, and snow cover data can be valuable in the grazing management of husbandry communities.

## Figures and Tables

**Table 1:** Linear mixed models for the effect of interactions between WST, SCD, SNC, and SOC on SOS of Inner Mongolia grassland. SOS: the start date of plant growth season; WST: average winter–spring air temperature; SCD: snow cover days; SNC: soil nitrogen content; and SOC: soil organic carbon content.

Response Variable	Explanatory Variables	AIC	*F*	*p*
	Grassland type × WST × SCD	2473.1	83.4	<0.001
	Grassland type × WST	2541.3	78.7	<0.001
	WST × SCD	2556.4	24.9	<0.05
SOS	Grassland type × WST × SNC	2582.1	68.3	<0.001
	WST	2594.2	18.4	<0.05
	WST × SNC	2611.4	15.7	<0.05
	Grassland type × WST × SOC	2621.7	47.4	<0.001

**Table 2:** Multiple linear regression and simple linear regression models for SOS prediction in pastoral communities distributed in different types of grasslands of Inner Mongolia. (SOS: the start date of plant growth season; WST: average winter–spring air temperature; SCD: snow cover days; VIF: variance inflation factor. RMSE: root mean square error, and MAE: mean absolute error).

Grassland Types	Models	*R* ^2^	*p*	VIF	RMSE	MAE
	*SOS* = −11.884*WST* + 18.528	0.313	<0.001		4.877	3.734
Meadow grasslands	*SOS* = −0.399*SCD* + 135.983	0.425	<0.001		4.347	3.518
	*SOS* = −6.778*WST* − 0.305*SCD* + 79.873	0.506	<0.001	1.305	3.676	2.921
	*SOS* = −26.378*WST* − 53.866	0.491	<0.001		8.792	6.635
Typical grasslands	*SOS* = −0.420*SCD* + 155.220	0.355	<0.001		10.860	8.548
	*SOS* = −21.046*WST* − 0.279*SCD* + 5.146	0.628	<0.001	1.146	8.274	6.351
	*SOS* = −17.133*WST* + 17.848	0.422	<0.001		4.368	3.507
Desert grasslands	*SOS* = −0.282*SCD* + 126.849	0.295	<0.001		4.648	3.750
	*SOS* = −13.647*WST* − 0.178*SCD* + 47.640	0.529	<0.001	1.171	3.812	3.188

## Data Availability

Data are contained within the article.
